# Ist eine Impfpflicht gegen das Coronavirus nötig?

**DOI:** 10.1007/s10273-021-2852-2

**Published:** 2021-02-20

**Authors:** Thomas Wein

**Affiliations:** Institut für Volkswirtschaftlehre, Leuphana Universität Lüneburg, Universitätsallee 1, 21335 Lüneburg, Deutschland

**Keywords:** H12, D62, I18

## Abstract

An die Zulassung der SARS-CoV-2-Impfstoffe knüpft sich die Hoffnung, die Pandemie schnellstmöglich überwinden zu können. Befragungen zeigen jedoch eine begrenzte Impfbereitschaft, die für eine Herdenimmunität eventuell nicht ausreicht. Im vorliegenden Beitrag wird spieltheoretisch untersucht, wie die einzelne Impfentscheidung im Hinblick auf das Impfverhalten der anderen ausfällt. Es zeigt sich, dass sich impfen zu lassen keine dominate Strategie ist. Die Wahrscheinlichkeit sich impfen zu lassen, nimmt jedoch mit den langfristigen Kosten der Pandemie zu. Demnach könnte die Impfbereitschaft erhöht werden, wenn die Politik stärker über die Folgekosten aufklären würde. Dann könnte eine Impfpflicht vermieden werden.

## Abstract (en)

The end of the pandemic requires that SARS-CoV-2 vaccines be used. However, vaccination itself can lead to temporary adverse health effects and/or long-term damage. Vaccination initially represents a private good demanded by an individual cost-benefit calculus; however, it also creates positive externalities and thus too few individual incentives to vaccinate. Getting vaccinated is not a dominant rational strategy, neither in the overall population, nor among the old and the young, nor when the long-term costs of the pandemic are taken into account. It is all the more important to “price in” the long-term consequences of a lasting pandemic.

Das Coronavirus SARS-CoV-2 mit der damit verbundenen Gefahr der COVID-19-Erkrankung ist das beherrschende Thema. Mit der bevorstehenden bzw. bereits erfolgten Zulassung von SARS-CoV-2-Impfstoffen verbindet sich für sehr viele die Hoffnung, die Pandemie möglichst schnell zu überwinden. Solange noch keine wirksameren Medikamente als bisher zur Verfügung stehen, setzt das Ende der Pandemie voraus, dass diese Impfstoffe verwendet werden. Einerseits sollen Erkrankungen, insbesondere schlimme Verläufe einer COVID-19-Erkrankung, verhindert und andererseits die Verbreitung des Virus vermindert werden, um damit eine sogenannte Herdenimmunität zu erreichen. Impfungen selbst können jedoch auch zu temporären Gesundheitsbeeinträchtigungen[1] und/oder Langzeitschäden führen. Letzteres ist umso eher denkbar, je neuartiger der verwendete Impfstoff ist. Aus ökonomischer Sicht stellt die Verhinderung der Erkrankung ein Gut dar, welches nach einem individuellen Nutzen-Kosten-Kalkül nachgefragt wird, jedoch mit positiven technologischen externen Effekten verbunden ist bzw. ein öffentliches Gut darstellt. In beiden Fällen gäbe es zu geringe individuelle Anreize, um die gesellschaftlich optimale Impfrate zu erreichen. Ohne wirtschaftspolitische Eingriffe wie z.B. die Subventionierung von Impfungen oder eine Impfpflicht würde die Corona-Pandemie möglicherweise weitgehend ungehindert wie bisher fortbestehen, die Infektionszahlen würden nicht substanziell sinken, schwere Verläufe und virusbedingte Todesfälle wären weiter zu verzeichnen. Die Notwendigkeit für Abstands- und Hygieneregeln oder für temporäre Lockdowns bliebe bestehen. Zumindest teilweise würden somit individuelle Freiheitsrechte weiterhin beschnitten werden, das Sozialprodukt würde vermindert und staatliche Unterstützungszahlen für Betroffene blieben erforderlich. Letzteres würde die finanzielle Leistungsfähigkeit des Staates in Frage stellen.

## Ist eine Impfpflicht denkbar?

Mehrere Politiker:innen verschiedener Parteien haben eine Impfpflicht gegen SARS-CoV-2 grundsätzlich ausgeschlossen. So setzt Bundesgesundheitsminister Spahn (2020) (CDU) statt Impfpflicht auf Freiwilligkeit, um die notwendige Herdenimmunität zu erreichen. Der bayerische Ministerpräsident Söder (2020) (CSU) lehnt die Pflicht ebenfalls ab, sie sei doch eher ein „Gebot“. Bundesjustizministerin Lambrecht (2020) (SPD) geht noch weiter und lehnt eine faktische Impfpflicht „durch die Hintertür“ ab, wenn Nicht-Geimpften im gesellschaftlichen Leben Nachteile angedroht würden. Ständige Impfkommission, Deutscher Ethikrat und Leopoldina (2020) lehnten in einer gemeinsamen Stellungnahme im November 2020 eine undifferenzierte, allgemeine Impfpflicht ab, nur bei schwerwiegenden Gründen und für eine präzise differenzierte Personengruppe sei sie vorstellbar. Beschäftigte im ständigen Kontakt mit Hochrisikogruppen könnten hierunter fallen, falls die Impfung das einzig verbleibende Mittel sei. Augsberg (2020), Mitglied des Ethikrats und Jurist, betont im Einklang mit dem Ethikrat noch einmal die Ablehnung

1 Für den ersten Impfstoff gegen Corona wird von Erschöpfung, Fieber und Muskelschmerzen berichtet (Rößler, 2020).

**Prof. Dr. Thomas Wein** ist Professor am Institut für Volkswirtschaftslehre der Leuphana Universität Lüneburg.

einer generellen Impfpflicht, verweist aber darauf, dass verfassungsrechtlich für bestimmte Berufsgruppen eine Pflicht denkbar wäre. Nicht nur in Deutschland, sondern auch in anderen Ländern gibt es eine Impfpflichtdebatte gegen das Coronavirus. Für andere Krankheiten gibt es durchaus und unter besonderen Umständen eine solche Impfpflicht (o. V., 2020).

## Meinungsumfragen zur Impfbereitschaft

Wenige wissenschaftliche Studien liegen bisher zur Frage vor, wie groß die Bereitschaft der Bevölkerung ist, sich gegen SARS-CoV-2 zu impfen. Eine Gruppe von europäischen Gesundheitsökonom:innen (Neumann-Böhme et al., 2020) haben in einer repräsentativen Online-Umfrage im April 2020 in sieben europäischen Ländern (Dänemark, Deutschland, Frankreich, Italien (dort insbesondere die stark betroffene Region der Lombardei), Portugal, Niederlande und Vereinigtes Königreich) 7.664 Personen gefragt, ob sie sich gegen das Virus impfen lassen würden, wenn ein Impfstoff verfügbar wäre. Die Bereitschaft variiert zwischen 62 % in Frankreich und 80 % in Dänemark und dem Vereinigten Königreich, Deutschland liegt nahe bei dem niedrigen Wert von Frankreich. Vor allem Männer und ältere Bürger:innen tendieren eher zur Impfung. Für Israel wurden 1.941 anonyme Onlinefragebögen ausgewertet, die im März 2020 erhoben wurden (Dror et al., 2020). Daran nahmen Beschäftigte aus dem Gesundheitswesen sowie aus der allgemeinen Bevölkerung teil. Eine höhere Impfbereitschaft lag für medizinische Beschäftigte vor, die mit COVID-19-Patient:innen beruflich in Berührung gekommen waren, sowie für die allgemeine Bevölkerung. Dagegen war das medizinische Personal ohne Corona-Kontakt weniger zur Impfung bereit. Aus einer Zusatzbefragung im Sozio-ökonomischen Panel (SOEP), die im Juni und Juli 2020 durchgeführt wurde, folgt, dass sich 70 % der Befragten freiwillig impfen lassen würden, wiederum vor allem Ältere; 50 % der Antwortenden treten immerhin für eine Impfpflicht ein (Graeber et al., 2020). Der Verhaltensökonom und Unternehmensberater Gerhard Fehr ist äußerst skeptisch, dass ein ausreichender Grad an freiwilligen Impfungen zustande kommt. Die in Meinungsumfragen geäußerte potenzielle Bereitschaft zur Impfung sei keinesfalls mit der tatsächlichen Impfteilnahme gleichzusetzen – was auch bei anderen Fragen, die im Zusammenhang mit öffentlichen Gütern stehen, der Fall sei (Pennekamp, 2020).

## Impfpflicht – eine Frage der Verhältnismäßigkeit

Nach § 20, VI, S. 1 Infektionsschutzgesetz kann der Bundesgesundheitsminister durch Rechtsverordnung mit Zustimmung des Bundesrats anordnen, dass „bedrohte Teile der Bevölkerung an Schutzimpfungen oder anderen Maßnahmen der spezifischen Prophylaxe teilzunehmen haben, wenn eine übertragbare Krankheit mit klinisch schweren Verlaufsformen auftritt und mit ihrer epidemischen Verbreitung zu rechnen ist“. Insofern wäre grundsätzlich eine Impfpflicht gegen SARS-CoV-2 denkbar, da prinzipiell die gesamte Bevölkerung durch das Virus bedroht ist, schwere Verlaufsformen offenkundig sind und sich das Virus ohne Schutzmaßnahmen epidemisch verbreitet. Freilich ist zu prüfen, ob eine Impfpflicht mit Art. 2, II GG[2] vereinbar ist, was insbesondere eine Frage der Verhältnismäßigkeit sein dürfte (Tonti, 2020). Zur Prüfung der Verhältnismäßigkeit einer SARS-CoV-2-Impfung schlägt Savulescu (2020) einen weitverzweigten Algorithmus vor, der Prüffragen über die potenzielle Schwere einer COVID-19-Erkrankung, zur Effektivität und Sicherheit des jeweiligen Impfstoffs, zu den Vor- und Nachteilen der Impfpflicht sowie zu den Alternativen jenseits der Impfungen formuliert. In seiner sehr vorsichtigen Bewertung und vor dem Hintergrund seines zum Zeitpunkt der Veröffentlichung sehr unvollständigen Stand des Wissens über mögliche Impfkandidat:innen sieht er eher finanzielle Anreize zur Impfung als eine strikte Impfpflicht als verhältnismäßig an.

## Ökonomische Modelle zur Impfbereitschaft

In der ökonomischen Theorie ist die Impfpflicht geradezu ein Lehrbuchklassiker für einen wohlbegründbaren Eingriff in die individuelle Handlungsfreiheit, da die einzelnen Individuen nur den eigenen Nutzen der Impfung wahrnehmen und die Vorteile des Schutzes für andere vernachlässigen (positiver technologischer externer Effekt) bzw. der Schutz vor ansteckenden Krankheiten – aufgrund der fehlenden Rivalität im Konsum bzw. fehlenden Ausschließbarkeit von „Impfverweigernden“ von den Früchten einer Impfung – ein reines öffentliches Gut darstellt. Modelltheoretisch gibt es drei wegweisende Aufsätze, die deutliche Differenzen zwischen privaten und sozialen Anreizen für Impfungen herausarbeiten. Brito, Sheshinski und Intriligator (1991) zeigen in einem statischen Modell, bei dem Impflinge unterschiedliche Kosten der Impfung haben, dass eine freiwillige Entscheidung für oder gegen Impfung mit einer höheren Wohlfahrt verbunden ist, als alle zur Impfung zu zwingen; freilich erreicht eine Besteuerung der Impfverweigernden bzw. Subventionierung der Freiwilligen ein noch höheres Wohlfahrtsniveau. Francis (1997) dagegen arbeitet mit einem dynamischen Modell, in dem sich Individuen zu jeder Zeit entscheiden können, ob sie sich impfen lassen wollen oder nicht. Bei homogenen, unendlich lebenden Individuen, die sich bei einer Infektion von der Krankheit nicht wieder erholen, gibt es einen modell-endogenen Schwellenwert, bis zu

2 „Jeder hat das Recht auf Leben und körperliche Unversehrtheit. Die Freiheit der Person ist unverletzlich. In diese Rechte darf nur auf Grund eines Gesetzes eingegriffen werden.“

Abbildung 1
**Individuell rationale Impfentscheidung**

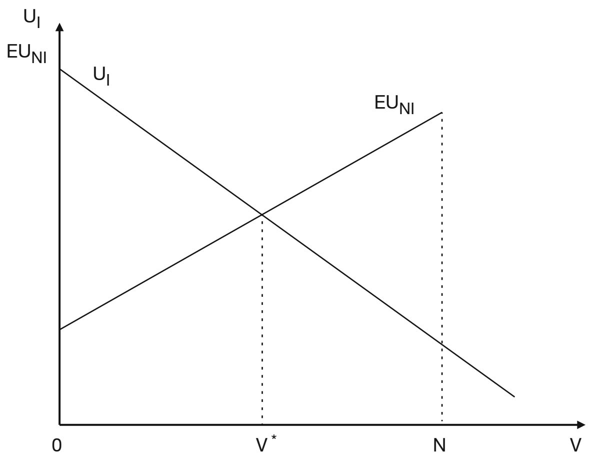

Quelle: Francis (1997, 387).

Abbildung 2
**Gesellschaftliche Kosten des Nicht-Impfens**

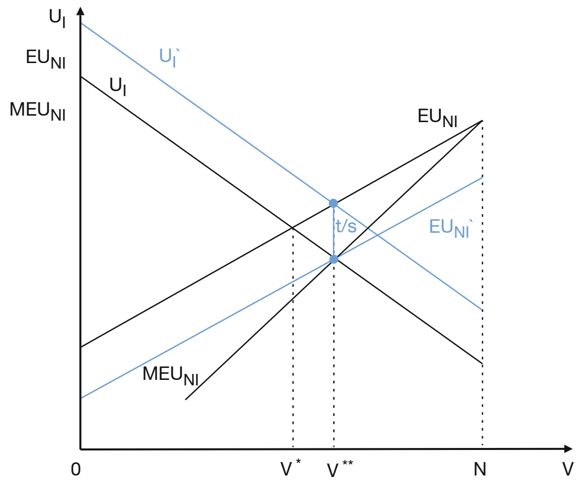

Quelle: In Anlehnung an Francis (1997, 389).

dem sich die Individuen der Impfung verweigern. Oberhalb des Schwellenwerts würden sich dann alle impfen lassen. In diesem sehr speziellen Modell entspricht die individuell rationale Impfneigung der gesellschaftlich optimalen, es bleibt kein Platz für eine Impfpflicht. Chen und Toxvaerd (2014) verallgemeinern die Ergebnisse von Francis, indem sich z. B. die Individuen von der Krankheit erholen können oder Impfstoffe unvollständig wirksam sein können.

## Die Ökonomik des Impfens

Zunächst wird anhand der grafischen Variante des statischen Impfmodells die „Ökonomik des Impfens“ verdeutlicht. Nach Francis (1997, 386-389) wird eine gegebene Bevölkerung N unterstellt, wobei sich die Bewohner:innen nur in ihren Kosten des Impfens unterscheiden. Je mehr Menschen sich impfen lassen, umso höher ist der erwartete Nutzen der Nicht-Geimpften. Im Falle von SARS-CoV-2 müssten die neuen Impfstoffe dafür sorgen, dass die Ansteckungsrisiken für Nicht-Geimpfte zurückgehen, was für die bisher bekannten Impfstoffe unklar ist. Für die grafische Darstellung wird vereinfachend unterstellt, dass die Menschen in absteigender Reihenfolge ihrer Impfkosten geimpft werden, d. h. Personen mit niedrigen Impfkosten werden früher geimpft als Menschen mit hohen Impfkosten. In Abbildung 1 wird auf der horizontalen Achse die Zahl der geimpften Personen V abgetragen. Je mehr Menschen geimpft werden, umso größer wird der erwartete Nutzen der Nicht-Geimpften ($EU_{NI}$) aufgrund des abnehmenden Risikos der Ansteckung. Aufgrund der unterschiedlichen Impfkosten (und der absteigenden Impfreihenfolge) fällt der sichere Nutzen der Geimpften ($U_I$). Für beide Kurven wird vereinfachend ein linearer Verlauf unterstellt. Im Bereich zwischen 0 und V* überwiegt der sichere Nutzen des Impfens den erwarteten Nutzen des Nicht-Impfens; folglich werden sich die Personen in diesem Bereich impfen lassen. Für die Individuen zwischen V* und N sind die erwarteten Nutzen des Nicht-Impfens größer als die sicheren Nutzen des Impfens; diese Individuen werden zu „Impfgegner:innen“.

Nimmt die Bevölkerung hohe Kosten des Impfens wahr, verschiebt sich die Kurve $U_I$ nach links unten. Im Extremfall liegt $U_I$ für alle unterhalb $EU_{NI}$, es wird sich niemand impfen lassen. Bei einem sehr hohen Ansteckungsrisiko für alle (homogenen) Individuen fällt der erwartete Nutzen bei Nicht-Impfung sehr gering aus. Die Kurve $EU_{NI}$ wird nach rechts unten verschoben. Im Extremfall lassen sich alle impfen (V* = N). Herdenimmunität, wonach der Virus keine ansteckbaren „Wirte“ mehr findet und deshalb „ausstirbt“, ist in diesem Modell mit identischen individuellen Ansteckungsrisiken nicht enthalten.

Gesamtgesellschaftlich sind diese Impfentscheidungen der Individuen jedoch verzerrt, da die Impfgegner:innen nicht berücksichtigen, dass ihre Entscheidung gegen das Impfen Rückwirkungen auf das Verhalten der anderen Individuen hat: Wenn sie sich nicht impfen, lohnt es sich ceteris paribus für die anderen mehr, sich zu impfen, da das Ansteckungsrisiko größer wird. Nur wenn sich alle impfen (V = N), entfällt dieser Effekt. Grafisch gibt es somit eine geringere soziale Grenznutzenfunktion des Nicht-Impfens ($MEU_{NI}$) als die individuelle $EU_{NI}$. In Abbildung 2 wird deutlich, dass das gesellschaftlich optimale Ausmaß der Impfung höher liegt, nämlich bei V**. Wirtschaftspolitisch kann man natürlich über eine Impfpflicht bis zu V** korrigierend eingreifen, oder analog zur Pigou-Lösung (Schmidt, 2019) das Nicht-Impfen mit der Steuer t bestrafen ($EU_{NI}$`) oder das Impfen mit einer Subvention s belohnen ($U_{NI}$`).

## Gesellschaftliche Kosten des Nicht-Impfens

Gerade im Falle der Pandemie durch SARS-CoV-2 sind neben den individuellen Kosten des Nicht-Impfens noch weitere, gesellschaftliche Kosten zu berücksichtigen. Diese schwer zu messenden Kosten sind im Wesentlichen:

- Einhaltung der AHA-L-Regeln: Abstand halten, Hygienemaßnahmen beachten, Alltagsmasken tragen und auf regelmäßiges Lüften geschlossener Räume achten. Die AHA-L-Regeln erzeugen Disnutzen in vielfältigen Formen: geringeres Gefühl der Nähe, Zeitkosten, erschwerte Kommunikation, Gefühl der Beeinträchtigung in der persönlichen Lebensführung, Kälte und Heizkosten. Diese Kosten sind sicherlich individuell verschieden und werden im Zeitablauf möglicherweise verstärkt wahrgenommen. Sie treten mit Sicherheit auf, sind individuell meist relativ niedrig, aber aufsummiert über die gesamte Bevölkerung durchaus bedeutsam.

- Solange sich SARS-CoV-2 weiterverbreitet, kommt es zu schweren Verläufen an COVID-19-Erkrankungen, die auch zu Sterbefällen führen. Manche Erkrankte leiden an Langfristbeschwerden (Long-COVID), deren Tiefe und Dauerhaftigkeit bisher nicht abgeschätzt werden kann. Eine Monetarisierung der fortbestehenden Krankheits- und Sterbefallrisiken ist schwierig, nichtsdestotrotz sind sie gesellschaftlich existent. Statistisch ist die Schwere der Erkrankung sicherlich alterskorreliert oder steht im Zusammenhang mit Vorerkrankungen/Vorbelastungen. Aus der Sicht des Einzelnen treten die Erkrankungen und damit die Kosten trotz allem unter Unsicherheit auf, da man nicht weiß, ob man sich infizieren und wie schwer man erkranken wird.

- Mit zunehmendem Infektionsgeschehen erreicht das Gesundheitssystem seine Grenzen, apparative Voraussetzungen der Intensivbehandlung wie Betten und Beatmungsgeräte fehlen und personale Kapazitäten erschöpfen sich bzw. sind nur begrenzt erweiterbar. Gesamtwirtschaftlich sind diese Zusatzkosten der Überforderung im Gesundheitswesen nicht zu vernachlässigen.

- Droht das Infektionsgeschehen außer Kontrolle zu geraten, sind (Teil-)Lockdowns unvermeidlich: Einzelhandelsgeschäfte werden geschlossen. Kulturveranstaltungen müssen abgesagt werden. Cafés und Restaurants dürfen keine Speisen und Getränke am Tisch verkaufen etc. Insbesondere Dienstleistungen des Freizeitkonsums werden am schnellsten geschlossen, da sie eher als verzichtbar gelten. Auf der Angebotsseite entstehen Einkommensverluste, die zumindest teilweise der (deutsche) Staat zu kompensieren versucht. Auf der Nachfrageseite entgeht Nutzen aus dem Freizeitkonsum, allerdings entfallen meist auch die entsprechenden Ausgaben. Güter des stationären Einzelhandels werden vielfach online beschafft; lokale Anbieter:innen verlieren, und manche werden mittelfristig zur Geschäftsaufgabe gezwungen. Wirtschaftlich noch kostspieliger wird es, wenn die Industrieproduktion stockt, sei es wegen zusammenbrechender Lieferketten oder coronabedingter Nachfrageschwäche. Beschäftigte werden temporär oder dauerhaft nicht mehr gebraucht, Kurzarbeitergeld muss bezahlt werden, oder es kommt zu Entlassungen mit Bezug von Arbeitslosengeld. Gesamtwirtschaftlich erleiden folglich die Haushalte und Unternehmen entweder Einkommens- und Nutzenverluste oder der Staat kompensiert sie, was durch erhöhte Kreditaufnahme finanziert wird. Erhöhte Kredite führen zu (gegenwärtig geringen) Zinszahlungen und zu späteren Rückzahlungsverpflichtungen, die aus dem Steueraufkommen der Zukunft zu begleichen sind. Bei einer wachsenden Wirtschaft mag die Rückführung der Schulden ohne Steuererhöhung finanzierbar sein, die Steuerzahlungen müssen trotzdem vom privaten Sektor erbracht werden. Die Lockdown-Kosten könnten überproportional ansteigen, wenn immer wieder Lockdowns fällig werden oder sie immer länger andauern müssen: Die wirtschaftliche Stabilität der Unternehmen gerät zunehmend unter Druck und die privaten Haushalte nehmen verstärkt die Einschränkungen des Lockdowns wahr.

Die genannten Kosten des Nicht-Impfens entstehen solange, wie das Infektionsgeschehen hoch bleibt. Erst bei „natürlicher“ Herdenimmunität würden all diese Kosten entfallen. Gegenüberzustellen sind natürlich immer noch die Kosten des Impfens, seien es die unsicheren kurzfristigen Impfbeschwerden oder die langfristig denkbaren, schwer abschätzbaren Impfschäden.

## Spieltheoretische Analyse

Im Folgenden werden anhand eines einfachen spieltheoretischen Modellrahmens in der Tradition der Debatte zur Bereitstellung öffentlicher Güter (Buchanan, 1968) Szenarien durchgespielt, um die zu erwartende Impfbereitschaft gegen das Coronavirus abzuschätzen. Betrachtet wird dabei die Entscheidungssituation eines Impflings, der sich impfen lässt oder sich einem Impfangebot verweigert. In einer einmaligen Entscheidungssituation bildet er Erwartungen, ob alle anderen potenziellen Impflinge, die zur gleichen Zeit eine Entscheidung treffen, sich impfen lassen werden oder nicht. Als risikoneutraler Impfling trifft er die Entscheidung, die seinen Erwartungswert maximiert. Für alle denkbaren Szenarien werden zwar willkürliche Zahlenwerte angenommen, die aber einigermaßen plausibel sein sollten.

Tabelle 1
**Allgemeine Impfanreize**

| Lasse ich mich impfen? | Lassen sich alle anderen impfen? | |
|---|---|---|
| | Ja | Nein |
| Ja | Nutzen:<br>- keine Erkrankung: +10<br>- Lockdown/AHA/Überforderung im Gesundheitswesen entfallen: +3<br>Kosten:<br>- Impfschaden: -1<br>Netto: +12 | Nutzen:<br>- keine Erkrankung: +10<br>Kosten:<br>- Impfschaden: -1<br>Netto: +9 |
| Nein | Nutzen:<br>- keine Erkrankung: +10<br>- Lockdown/AHA/Überforderung im Gesundheitswesen entfallen: +3<br>Netto: +13 | Netto: 0 |

Quelle: Eigene Darstellung.

Tabelle 2
**Impfanreize der Jüngeren**

| Lasse ich mich impfen? | Lassen sich alle anderen impfen? | |
|---|---|---|
| | Ja | Nein |
| Ja | Nutzen:<br>- keine Erkrankung: +0,5<br>- Lockdown/AHA/Überforderung im Gesundheitswesen entfallen: +3<br>Kosten:<br>- Impfschaden: -1<br>Netto: +2,5 | Nutzen:<br>- keine Erkrankung: +0,5<br>Kosten:<br>- Impfschaden: -1<br>Netto: -0,5 |
| Nein | Nutzen:<br>- keine Erkrankung: +0,5<br>- Lockdown/AHA/Überforderung im Gesundheitswesen entfallen: +3<br>Netto: +3,5 | Netto: 0 |

Quelle: Eigene Darstellung.

## Eine erste, sehr vereinfachende Einschätzung

In Tabelle 1 werden Nutzen- bzw. Kostenveränderungen abgetragen, ausgehend von der gegenwärtigen Situation. Entscheidet sich der Impfling für eine Impfung und alle anderen lassen sich impfen (vgl. Tabelle 1, links oben), soll er nicht mehr an COVID-19 erkranken können, die Pandemie wäre besiegt. Hier wird ein erwarteter Nutzen von +10 Geldeinheiten (GE) unterstellt, der sich aus den Vorteilen ergibt, sich nicht mehr infizieren zu können, nicht mehr schwer (auch mit Langzeitfolgen) zu erkranken oder gar zu versterben. Wenn sich alle impfen lassen, droht mit Sicherheit keine Überforderung des Gesundheitswesens mehr, AHA-L-Regeln und Lockdowns entfallen. Denkbar wäre daraus ein sicherer individueller Nutzen von +3 GE. Aus der individuellen Impfung folgen mögliche Impfbeschwerden und völlig unbekannte Langzeitrisiken, der erwartete Schaden wird auf -1 GE geschätzt. Netto ergibt sich somit im Feld links oben ein erwarteter Nutzen von +12 GE. Impft sich der Einzelne nicht und alle anderen lassen sich impfen (links unten), wird der Einzelne ebenfalls nicht mehr erkranken, das Gesundheitswesen wird nicht mehr überfordert, AHA-L-Regeln und Lockdowns entfallen. Wer sich nicht impfen lässt, muss nicht mit Impfbeschwerden oder Impfschäden rechnen, erwartete Schäden in Höhe von -1 GE entfallen. Netto erbringt links unten somit einen sicheren Nutzen von +13 GE. Lasse ich mich selbst impfen, aber alle anderen verzichten auf die Impfmöglichkeit (rechts oben), kann ich nicht mehr erkranken (+10 GE), aber die coronabedingten Einschränkungen und Überforderungen bleiben. Die erwarteten Impfschäden schlagen jedoch ebenfalls negativ zu Buche (-1 GE). Netto folgt daraus ein erwarteter Nutzen von +9 GE. Rechts unten schreibt den Status quo fort, niemand wird geimpft, Einschränkungen und Überforderungen bleiben, der Nettonutzen sei gleich Null. Erst wenn auf natürliche Weise Herdenimmunität eintritt, würde eine neue, bessere Situation eintreten. Entsprechend der Tabelle 1 ist es für den Einzelnen rational, sich nicht impfen zu lassen, wenn alle anderen sich impfen lassen (+13 GE > +12 GE), und sich impfen zu lassen, wenn alle anderen sich nicht impfen lassen (+9 GE > 0 GE). Es gibt also keine eindeutige Antwort (keine dominante Strategie). Lässt man jedoch Erwartungen über die Wahrscheinlichkeiten zum Verhalten der anderen zu, kann man eine kritische Wahrscheinlichkeit p für die unbedingte individuelle Impfbereitschaft berechnen: Der Erwartungswert für Impfen (12p + (1-p) 9) muss größer sein als der Wert für Nicht-Impfen (13p + (1-p) 0), was bei einer Impfwahrscheinlichkeit unter 90 % der anderen erfüllt ist. Laut Tabelle 1 ist somit das Freifahrerverhalten bei Impfung denkbar.

## Anreize bei Jung und Alt?

Die Darstellung wird realitätsnäher, wenn man zwischen Älteren und Jüngeren unterscheidet (vgl. Tabellen 2 und 3). In Tabelle 2 wird die Auszahlungsmatrix eines Jüngeren dargestellt. Aufgrund seines relativ geringen Alters weist er ein sehr geringes Erkrankungsrisiko auf. Der erwartete Nutzen einer Impfung beträgt daher nur +0,5 GE. Hier ergeben sich in drei der vier Fälle veränderte Nettonutzenwerte. Allerdings weist dieser junge Mensch immer geringere Nutzenwerte für die individuelle Nicht-Impfung auf im Vergleich zur Impfung. Folglich wird er sich nicht impfen lassen, unabhängig vom Verhalten der anderen (dominante Strategie).

Ältere Menschen haben ein deutlich höheres Erkrankungsrisiko und gewinnen damit mehr, wenn sie sich impfen lassen (+19 GE) (vgl. Tabelle 3). Gemäß der veränderten Nettowerte ist ihr Verhalten weiterhin jedoch davon abhängig, wie sich alle anderen verhalten: Lassen sich alle anderen impfen, hat die Nicht-Impfung immer noch einen relativen Vorteil, da sie dann die individuellen Kosten des

Tabelle 3
**Impfanreize der Älteren**

| | | Lassen sich alle anderen impfen? | |
|---|---|---|---|
| | | Ja | Nein |
| Lasse ich mich impfen? | Ja | Nutzen:<br>- keine Erkrankung: +19<br>- Lockdown/AHA/Überforderung im Gesundheitswesen entfallen: +3<br>Kosten:<br>- Impfschaden: -1<br>Netto: +21 | Nutzen:<br>- keine Erkrankung: +19<br>Kosten:<br>- Impfschaden: -1<br>Netto: +18 |
| | Nein | Nutzen:<br>- keine Erkrankung: +19<br>- Lockdown/AHA/Überforderung im Gesundheitswesen entfallen: +3<br>Netto: +22 | Netto: 0 |

Quelle: Eigene Darstellung.

Tabelle 4
**Überforderung der Gesellschaft**

| | | Lassen sich alle anderen impfen? | |
|---|---|---|---|
| | | Ja | Nein |
| Lasse ich mich impfen? | Ja | Nutzen:<br>- keine Erkrankung +10<br>- Lockdown/AHA/Überforderung im Gesundheitswesen entfallen: +3<br>Kosten:<br>- Impfschaden: -1<br>Netto: +12 | Nutzen:<br>- keine Erkrankung +10<br>Kosten:<br>- Impfschaden: -1<br>- Überforderung von Gesellschaft und Staat: -4<br>Netto: +5 |
| | Nein | Nutzen:<br>- keine Erkrankung: +10<br>- Lockdown/AHA/Überforderung im Gesundheitswesen entfallen: +3<br>Netto: +13 | Kosten:<br>- Überforderung von Wirtschaft und Staat: -4<br>Netto: -4 |

Quelle: Eigene Darstellung.

Nicht-Impfens nicht tragen müssen (+22 > +21); erwarten sie, dass sich alle anderen nicht impfen lassen, überwiegen die individuellen Impfvorteile (+18 > 0). Die kritische Wahrscheinlichkeit für die Impfbereitschaft der anderen steigt in diesem Falle an: Nur wenn sich alle anderen mit einer Wahrscheinlichkeit von weniger als 95 % impfen lassen, lasse ich mich auch impfen.

### Überforderung bei anhaltender Pandemie

Die bisher angenommenen Auszahlungen sind möglicherweise zu optimistisch, da sie die langfristigen Folgen des Status quo unterschätzen. Bei länger anhaltender Pandemie mit weiteren Lockdowns wird das Sozialprodukt deutlich reduziert, bisher funktionierende Geschäftsmodelle sind nicht mehr rentabel, Arbeitslosigkeit nimmt stark zu, der Staat kann die finanziellen Folgen der Pandemie zunehmend nicht mehr ausgleichen, und nicht zuletzt empfindet die Bevölkerung die individuellen Freiheitsbeschränkungen als große Last. In Tabelle 4 kommen in der rechten Spalte Überforderungskosten von Gesellschaft und Staat in Höhe von +4 GE dazu. Der Nettonutzen des Einzelnen, der sich impfen lässt, sinkt auf +5 GE, dessen der sich der Impfung verweigert, geht auf -4 GE zurück. Weiterhin gilt zwar, dass der Einzelne sich nicht impfen lässt, wenn er erwartet, dass alle anderen sich impfen lassen (+13 GE > +12 GE) und der Einzelne sich impfen lässt, wenn alle anderen sich nicht impfen lassen (+5 GE > -4 GE). Da jedoch die Überforderungskosten auch die Jüngeren treffen, dürfte auch deren Impfbereitschaft ansteigen.

## Wirtschaftspolitische Schlussfolgerungen

Vonseiten der Politik und Teilen der Wissenschaft wird eine allgemeine Impfpflicht abgelehnt. Rechtlich ist eine Impfpflicht gegen das SARS-CoV-2-Virus gemäß § 20 VI des Infektionsschutzgesetzes durchaus denkbar, allerdings müsste dieser Eingriff in die körperliche Unversehrtheit (Art. 2 II GG) verhältnismäßig sein. Je gravierender die Folgen von COVID-19 für Erkrankte wären und je mehr Nachteile Wirtschaft und Gesellschaft durch eine dauerhafte Pandemiebekämpfung ohne Impfung hinnehmen müssten, umso eher wäre eine Pflicht verhältnismäßig. Auch muss in die Verhältnismäßigkeitsprüfung eingehen, welche Impfbeschwerden und -schäden drohen bzw. welche Alternativen zur Virusbekämpfung zur Verfügung stehen. Befragungsergebnisse zeigen eine begrenzte Impfbereitschaft, wohl eher bei Älteren. Skepsis ist aber angebracht, ob diese Bereitschaft auch noch gilt, wenn die Impfung bevorsteht.

Die ökonomischen Modelle des Impfens zeigen, dass Impfen mit positiven externen Effekten verbunden sein kann und deswegen gesellschaftlich gesehen zu wenige Impfungen stattfinden. Je höher die individuellen Kosten des Impfens, umso eher würde zu wenig geimpft. Gerade bei der COVID-19-Pandemie stehen aber weitere vielfältige, schwerwiegende gesellschaftliche Kosten im Raum, wenn die Pandemie ohne hinreichende Impfneigung nicht endet. Die einfachen, sicherlich begrenzten spieltheoretischen Modelle mit beispielhaften Auszahlungen zeigen, dass Sich-Impfenlassen keine dominante rationale Strategie ist, weder in der Gesamtbevölkerung, noch bei Alten und Jungen, noch bei Berücksichtigung der langfristigen Kosten der Pandemie. Für Junge ist es nie rational, sich impfen zu lassen, zumindest wenn man Risikoneutralität unterstellt. Bezieht man die Überforderungskosten einer lang anhaltenden Pandemie ein, dürfte die Impfbereitschaft allgemein steigen. Die Impfpflicht ist eine rigorose Methode, um positive Externalitäten zu internalisieren.

Aus der Umweltökonomik weiß man (Menges, 2019), dass diese Form der Auflagenlösung insbesondere dann sinnvoll ist, wenn alle Emittenten kostenmäßig gleich sind. Für das Coronavirus könnte dies durchaus gegeben sein, was sich möglicherweise auch durch eine differenzierte juristische Verhältnismäßigkeitsprüfung ergeben würde. Denkbar sind natürlich auch (finanzielle) Anreize für das Impfen und (finanzielle) Sanktionen bei Nicht-Geimpften. Vermutlich dauert es in Deutschland zu lang, bis die politische und juristische Diskussion pro Impfpflicht oder für (finanzielle) Anreize zum Ende kommt. Die Politik könnte aber bereits jetzt der Bevölkerung die finanziellen Folgen der vermutlich notwendigen Rettungsaktionen deutlich machen, z.B. notwendige Kürzungen oder Steuererhöhungen skizzieren. Dies könnte sogar auf der individuellen Ebene die Bereitschaft erhöhen, die Risiken der Impfung hinzunehmen. Insofern wäre eine solche Klarheit eine Maßnahme, um der bereits seit langem bekannten Fiskalillusion entgegenzuwirken (Oates, 1985). All die genannten Punkte setzen jedoch voraus, dass die Impfung gegen das SARS-CoV-2-Virus (vollständigen) Infektionsschutz für andere vermittelt.
